# Interpretable two-stage deep learning for pediatric obstructive sleep apnea diagnosis using lateral cephalograms

**DOI:** 10.3389/fped.2026.1817094

**Published:** 2026-04-21

**Authors:** Jiayi Zhang, Jiao Tan, Xuesha Tong, Huiya Wang, Yue Zhao, Jinlin Song, Yang Liu

**Affiliations:** 1 The Affiliated Stomatological Hospital of Chongqing Medical University, Chongqing, China; 2 Chongqing Key Laboratory of Oral Diseases, Chongqing, China; 3 Chongqing Municipal Key Laboratory of Oral Biomedical Engineering of Higher Education, Chongqing, China; 4 Chongqing Municipal Health Commission Key Laboratory of Oral Biomedical Engineering, Chongqing, China; 5 School of Communication and Information Engineering, Chongqing University of Posts and Telecommunications, Chongqing, China; 6 Western Institute of Digital-Intelligent Medicine, Chongqing, China

**Keywords:** artificial intelligence, deep learning, lateral cephalogram, obstructive sleep apnea hypopnea syndrome, pediatric

## Abstract

**Objective:**

Obstructive sleep apnea-hypopnea syndrome (OSAHS) is a prevalent sleep-breathing disorder in pediatrics, yet early and accurate detection remains challenging due to the limited accessibility and efficiency of conventional diagnostic methods. This study aims to develop and validate an artificial intelligence framework that utilizes routine lateral cephalograms (LCs) to provide automated, accurate, and interpretable risk evaluations for pediatric OSAHS.

**Methods:**

We retrospectively enrolled 188 children from two hospitals between January 2021 and October 2025. A total of 150 LCs were used for cross- validation, and 38 LCs were reserved as an independent test dataset. Using LCs, we proposed and developed an interpretable two-stage framework for pediatric OSAHS diagnosis. The first stage segmented the upper airway, and the second stage performed classification using a modified fusion model that integrate information from both craniofacial structures and the upper airway. We compared different input strategies and used Grad-CAM for model interpretation. Clinical utility was evaluated in a reader study comparing dentists' performance across different experience levels.

**Results:**

The upper airway segmentation achieved a mean DSC of 0.931 and an IoU of 0.872. For OSAHS classification, the fusion model achieved an AUC of 0.945 (95% CI: 0.863–0.994) and an F1 score of 0.933 (95% CI: 0.818–0.995), outperforming the LCs model (AUC 0.797, 95% CI: 0.585–0.968) and the mask-based ROI model (AUC 0.882, 95% CI: 0.748–0.983). Grad-CAM consistently highlighted anatomically plausible regions related to craniofacial structure and the upper airway. In the reader study, AI assistance increased diagnostic accuracy by 0.165 for junior dentists and 0.237 for senior dentists.

**Conclusion:**

Our model represents a promising tool for automated pediatric OSAHS diagnosis based on routinely acquired LCs in dental settings. By enhancing diagnostic accuracy and interpretability, it has the potential to support early detection and individualized management.

## 1 Introduction

Pediatric obstructive sleep apnea-hypopnea syndrome (OSAHS) is a sleep-breathing disorder characterized by intermittent partial or complete obstruction of the upper airway during sleep, affecting 1%–4% of children globally (1–3). It is associated with various comorbidities including craniofacial developmental abnormalities, cognitive and neurological impairments, and behavioral and learning deficits (4–7). In addition, affected children often require substantially greater healthcare utilization than their healthy peers, placing a heavy burden on families and healthcare systems (8, 9). Although polysomnography (PSG) remains the diagnostic gold standard, its high cost, limited accessibility, and labor-intensive workflow restrict its use as a large-scale screening tool in children (3, 10, 11). Hence, a rapid, convenient diagnostic approach is urgently needed to enable early screening and timely intervention for early OSAHS.

Lateral cephalograms (LCs) are routinely acquired in dental and orthodontic practice with low radiation exposure and broad availability (12, 13). They provide information on both craniofacial structures and the upper airway, which are closely related to pediatric OSAHS, as the disease is associated not only with airway narrowing but also with craniofacial morphological alterations such as mandibular retrusion, maxillary deficiency, and vertical skeletal disproportions (14–17). These imaging findings make LCs a potentially useful tool for opportunistic screening in routine dental settings. However, conventional cephalometric assessment relies on manual measurements of a limited number of linear and angular parameters. It is time-consuming and subject to inter- and intra-observer variation (12, 13). In addition, LC images are two-dimensional and contain overlapping anatomical structures, making subtle upper airway patterns difficult to evaluate. These limitations reduce the utility of LCs for large-scale screening and detailed risk assessment.

In recent years, artificial intelligence (AI), particularly deep learning (DL) techniques, has shown considerable promise in automating the extraction of features from medical images and predicting disease risks (18–20). These advancements present a valuable opportunity to address the diagnostic challenges for OSAHS (21). DL, particularly convolutional neural networks (CNNs), has shown promise in identifying OSAHS from LCs in adults (22–24). However, pediatric OSAHS differs from adult disease in craniofacial development, pathophysiological mechanisms, and clinical presentation. These differences limit the direct transferability of adult-based models to children. Pediatric-specific models based on routine LCs remain scarce (25–27), emphasizing the urgent need for models that address the unique characteristics of children with OSAHS.

In this study, we propose an interpretable DL framework designed for pediatric OSAHS diagnosis using routinely acquired dental LCs. The framework combines automatic airway segmentation with image-based classification and integrates global craniofacial information with local airway features. We also used Grad-CAM to visualize the image regions contributing to model decisions. Our aim was to provide an automated and clinically relevant tool for pediatric OSAHS screening in routine dental settings.

## 2 Materials and methods

This retrospective study was approved by the Ethics Committee of the Affiliated Stomatological Hospital of Chongqing Medical University (approval number: CQHS-REC-2025, LS No. 126) and was performed in accordance with the principles of the Declaration of Helsinki (2013). Because data were collected retrospectively from LCs with no direct patient contact, the requirement for informed consent was waived. This study was reported in accordance with the Checklist for Reporting of Artificial Intelligence in Medical Imaging (CLAIM 2024) guidelines (Supplementary Table S1).

### 2.1 Patient data collection

**Figure 1:**
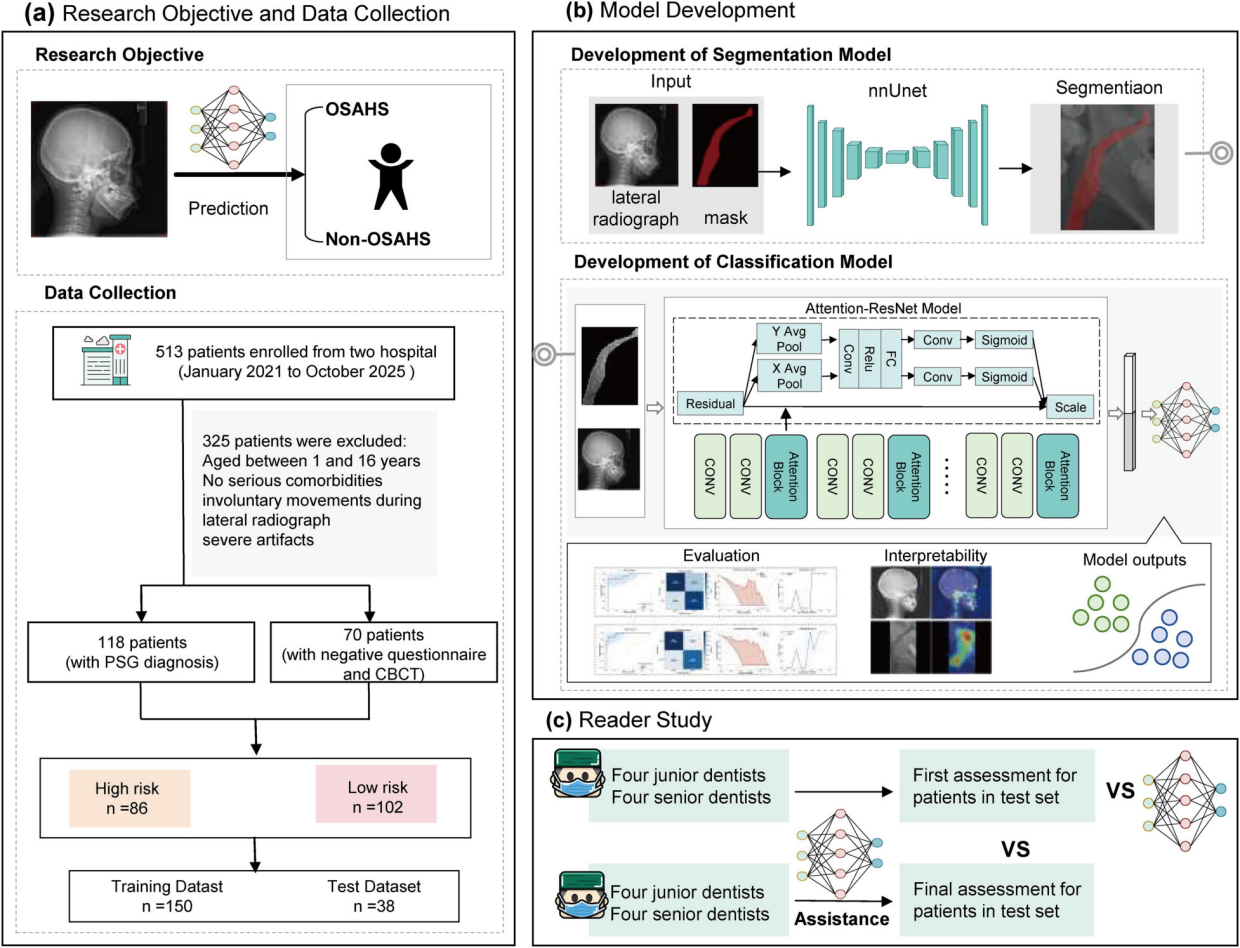
Overview of the study **(a)** research objective and data collection. **(b)** Model development: The model includes two networks: the segmentation model and the Attention-ResNet model. The model is trained using patient-level diagnostic labels and segmentation masks of LCs. **(c)** Reader study on the test dataset with and without AI assistance.

We retrospectively reviewed the LCs of 513 patients between January 2021 and October 2025 from the Ranjiaba and Shangqingsi campuses of the Affiliated Stomatological Hospital of Chongqing Medical University. After applying the exclusion criteria, data from 188 patients were included, as shown in Figure 1a. The apnea–hypopnea index (AHI) was used to assess the severity of sleep-disordered breathing, which is defined as the number of obstructive apneas, hypopneas, and mixed apneas per hour during sleep. Diagnostic classification followed: (1) Non-OSAHS: AHI <1/hr, (2) OSA: AHI ≥1/h (4). Baseline clinical and diagnostic information, including age, sex, and AHI were extracted from medical records. Among the included patients, 118 underwent PSG and had AHI results available. The remaining 70 patients were included in the non-OSAHS control group based on combined questionnaire screening using the Pediatric Sleep Questionnaire (PSQ) and OSA-18, supplemented by CBCT and clinical evaluation (14, 28, 29). Specifically, these patients were required to show no obvious adenoidal or tonsillar hypertrophy, a patent nasal airway, and no apparent upper-airway narrowing on CBCT, with no documented clinical history suggestive of sleep-disordered breathing. A representative Non-OSAHS control case is shown in Supplementary Figure S1. The dataset was first divided into a development set (80%) and an independent hold-out test set (20%). Five-fold cross-validation was performed exclusively within the development set, while the independent test set was used for final evaluation.

### 2.2 Image acqusition and annotation

LCs were obtained using a Kodak 9,000× imaging machine (25.7 cm × 25.6 cm). All x-rays were taken at the end of exhalation during the breathing cycle, with strict standardization of head and jaw positioning to obtain the clearest soft tissue images of the oropharynx. Two dentists manually annotated the regions of interest (ROI) using the open-source software Labelme (version 5.0.1; Computer Science and Artificial Intelligence Laboratory, MIT, Cambridge, MA, USA). One month after the initial annotation, 20 patients were randomly selected, and the same dentists redefined the ROIs to assess intra-rater reliability, using the Intraclass Correlation Coefficient (ICC).

### 2.3 Segmentation model

We used the 2D full-resolution nnU-Net framework to segment the upper airway on LCs (30). It follows an encoder-decoder architecture and integrates multi-scale image features for medical image segmentation. The segmentation model takes LC images as the input and outputs the segmentation map of upper airway. The model was initialized with random weights and trained from scratch for 150 epochs using the stochastic gradient descent (SGD) optimizer. A combination of dice and cross-entropy loss functions was employed during training. The initial learning rate was set to 1 × 10^−2^ with a weight decay of 3 × 10^−5^, and a momentum parameter was set to 0.99. A polynomial decay schedule was used for learning rate adjustment.

### 2.4 Classification models

#### 2.4.1 Single-input strategies

We constructed three image input strategies for classification. The first used the full LC image and preserved the complete craniofacial context. The second used a bounding-box ROI image generated from the predicted airway mask. This strategy cropped the airway-centered region while retaining adjacent surrounding tissues. The third used a mask-based ROI image. We evaluated these three input strategies separately in the classification task.

#### 2.4.2 Backbone selection and modified ResNet-34

To determine the optimal feature extraction backbone, we initially explored several architectures for the classification task, including AlexNet, ResNet-18, ResNet-34 (31, 32). We initialized all models with ImageNet-pretrained weights and fine-tuned them under the same data split and training setting. In our preliminary experiments for pediatric OSAHS classification based on LC images, ResNet-34 achieved the best performance. We therefore selected ResNet-34 as the backbone of the final framework. Additionally, we integrated the Coordinate Attention (CoordAtt) module into the model to enhance feature extraction capabilities, allowing the model to better capture the anatomical features of the upper airway (33). It encodes long-range dependencies along the horizontal and vertical directions while preserving positional information. In the modified ResNet-34, we inserted CoordAtt into each residual block, after the second convolutional layer and batch normalization, and before addition with the identity shortcut branch.

#### 2.4.3 Fusion model

We evaluated the full LC image, the bounding-box ROI image, and the mask-based ROI image separately. The comparison showed that the full LC image preserved richer global craniofacial information, whereas the mask-based ROI image captured local airway morphology more effectively. Based on these findings, we built the final fusion model using the full LC image and the mask-based ROI image. The two input streams were processed by two ResNet-34-CoordAtt classifiers, and each branch produced a 512-dimensional feature vector. We concatenated feature vectors along the feature dimension to generate a 1,024-dimensional fused representation. We then standardized this fused representation with StandardScaler and used it as input to a Multi Layer Perceptron (MLP) classifier. The MLP consisted of three fully connected layers with ReLU activation applied after the first two layers.

#### 2.4.4 Training configuration

For classification, we resized all input images to 224 × 224 pixels and normalized them using the ImageNet mean and standard deviation. We applied data augmentation to improve model robustness and mitigate overfitting in this relatively small cohort. The augmentation pipeline included random affine transformation with rotations up to ±7°, translations up to 4% in both horizontal and vertical directions, scaling in the range of 0.92–1.08, and random brightness and contrast perturbation. We trained all classification models with the AdamW optimizer, with an initial learning rate of 1 × 10^−4^, a weight decay of 1 × 10^−2^, and 150 epochs with a batch size of 8. The loss function in single-input classifiers was cross-entropy loss and fusion model was BCEWithLogits loss. During training, we monitored AUC in each fold and retained the model checkpoint with the highest AUC.

All experiments were implemented in Python 3.10.15 and ran the models on an NVIDIA GeForce RTX 4090D GPU with 24 GB of memory.

### 2.5 Interpretability

Two representative cases were selected from the OSA and Non-OSAHS groups. Gradient-weighted class activation mapping (Grad-CAM) was utilized to highlight important ROIs for classification targets (34). It was applied to the last convolutional layer of the modified ResNet-34 backbone. The resulting class activation maps were used to inspect whether highlighted regions corresponded to clinically plausible anatomy related to airway narrowing and craniofacial structure.

### 2.6 Reader study

We conducted a reader study with two groups dentists of varying experience levels: four junior dentists with less than one year of experience and four senior dentists with more than 10 years of experience. After obtaining informed consent, each of the 8 participants independently evaluated 38 LCs from the test set. To simulate real clinical diagnostic scenarios, only the patients’ age and gender were disclosed, while other clinical details were concealed during the evaluation process. The test set was reassessed in a random order one week later with AI assistance.

### 2.7 Performance evaluation metrics

We evaluated segmentation performance using the Dice Similarity Coefficient (DSC), Intersection over Union (IoU), precision, recall, and F1 score. We further assessed boundary agreement using the 95th percentile Hausdorff distance (HD95) and the average surface distance (ASD). For classification performance, we used the area under the receiver operating characteristic curve (AUC), accuracy, sensitivity, specificity, and F1 score.

$$\begin{array}{rl} & \mathrm{DSC}=\frac{2\times TP}{2\times TP+FP+FN}\\ & \mathrm{IoU}=\frac{TP}{TP+FP+FN}\\ & \mathrm{Accuracy}=\frac{TP+TN}{TP+TN+FP+FN}\\ & F1-Score==\frac{2\times TP}{2\times TP+FP+FN}\\ & \mathrm{Sensitivity}=\frac{TP}{TP+FN}\\ & \mathrm{Specificity}=\frac{TN}{TN+FP} \end{array}$$

## 3 Results

### 3.1 Demographic characteristics

This study included 188 pediatric patients, split into two datasets: a cross-validation dataset (*n* = 150) and a test dataset (*n* = 38). The demographic characteristics of the patients are summarized in Table 1. A total of 150 pediatric patients were included in the cross-validation dataset, with a mean age of 10.61 ± 3.27 years. Of these, 46.0% were diagnosed with OSAHS, and 54.0% were classified as Non-OSAHS. While in test dataset, the mean age was 10.55 ± 3.01 years, with 44.7% of patients diagnosed with OSAHS, and 55.3% were non-OSAHS.

**Table 1:** Demographic characteristics of patients.

Variable	Cross-validation (*n* = 150)	Test (*n* = 38)
Age, year	10.61 ± 3.27	10.55 ± 3.01
Gender		
Male	81 (54.0%)	22 (57.9%)
Female	69 46.0%)	16 (42.1%)
Classification		
OSAHS	69 (46.0%)	17 (44.7%)
Non-OSAHS	81 (54.0%)	21 (55.3%)

### 3.2 Segmentation performance evaluation

**Figure 2:**
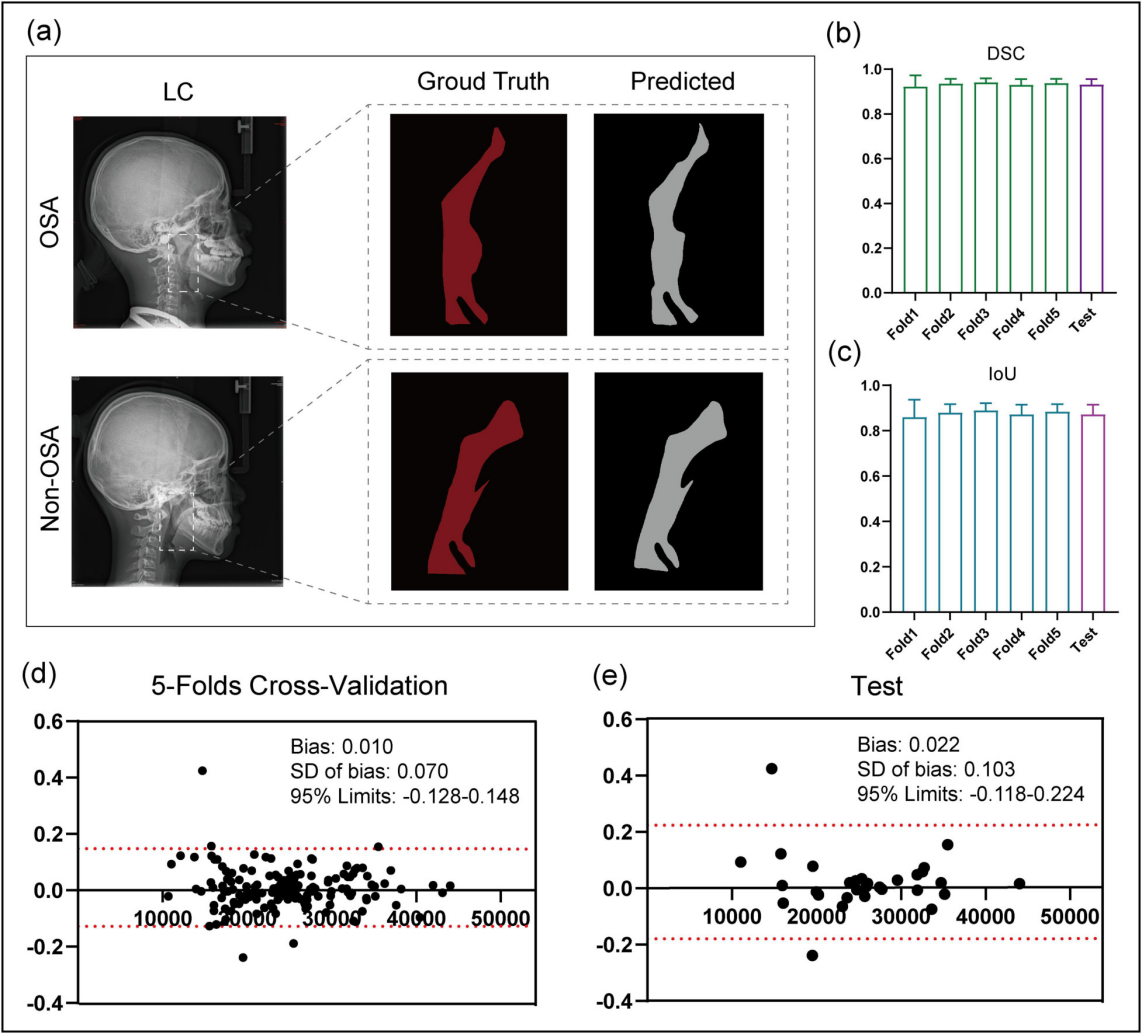
Segmentation performance of upper airway. **(a)** Visualization and ground truth comparison of segmentation results for two cases **(b)** the mean DSC of the upper airway across 5-folds validation and test dataset. **(c)** The mean IoU of the upper airway across 5-folds validation and test dataset. **(d)** The Bland-Altman of surface of upper airway in across 5-folds validation dataset. **(e)** The bland altman of surface of upper airway in across test dataset.

Table 2 and Figure 2 present the evaluation results of the segmentation performance. Figure 2a shows the original images, ground truth, and segmentation masks from nnUNet for two representative cases (OSAHS and Non-OSAHS). In upper airway segmentation, the model consistently achieved a DSC of 0.934, IoU of 0.877, precision of 0.930, recall of 0.939, and F1 score of 0.934 in 5-fold cross-validation. On the independent test dataset, the model achieved a DSC of 0.931, IoU of 0.872, precision of 0.933, recall of 0.931, and F1 score of 0.931. In addition, the model achieved an HD95 of 15.0 and an ASD of 4.0, indicating good overall contour agreement. As shown in Figures 2d,e, Bland–Altman analysis demonstrated that the mean area deviation of the predicted ROI was 0.010 in the cross-validation set and 0.022 in the independent test set. Overall, the segmentation model achieved satisfactory performance in delineating the upper airway.

**Table 2:** Evaluation of the segmentation performance.

Dataset	DSC	IoU	Precision	Recall	F1-Score	HD95	ASD
Validation dataset	0.934	0.877	0.930	0.939	0.934	14.9	4.1
Test dataset	0.931	0.872	0.933	0.931	0.931	15.0	4.0

### 3.3 Evaluation of backbone architecture

Table 3 presents the performance evaluation of different backbone network models. The AlexNet model achieved an AUC of 0.815 (95% CI: 0.662–0.948), accuracy of 0.799 (95% CI: 0.675–0.925), and an F1 score of 0.804 (95% CI: 0.667–0.920). In comparison, the ResNet-18 model showed an improvement, with an AUC of 0.860 (95% CI: 0.717–0.976), accuracy of 0.842 (95% CI: 0.711–0.947), and an F1 score of 0.849 (95% CI: 0.706–0.957). The ResNet-34 model further enhanced the AUC to 0.886 (95% CI: 0.759–0.984), though its accuracy was slightly lower at 0.815 (95% CI: 0.684–0.921). Based on these results, we selected ResNet-34 as the backbone network and incorporated the CoordAtt module for further improvement. The modified model achieved an AUC of 0.882 (95% CI: 0.748–0.983), accuracy of 0.842 (95% CI: 0.711–0.947), F1 score of 0.838 (95% CI: 0.690–0.952), recall of 0.940 (95% CI: 0.818–1.00), precision of 0.762 (95% CI: 0.556–0.947), and specificity of 0.834 (95% CI: 0.607–0.971). The basic properties of the pretrained CNN backbones are summarized in Supplementary Table S3.

**Table 3:** Evaluation of backbones architectures.

Models	AUC (95% CI)	Accuracy (95% CI)	F1-Score (95% CI)	Sensitivity (95% CI)	Specificity (95% CI)	PR-AUC (95% CI)
AlextNet	0.815 (0.662–0.948)	0.799 (0.675–0.925)	0.804 (0.667–0.920)	0.808 (0.611–0.857)	0.788 (0.583–0.952)	0.776 (0.568–0.961)
ResNet-18	0.860 (0.717–0.976)	0.842 (0.711–0.947)	0.849 (0.706–0.957)	1.00 (1.00–1.00)	0.715 (0.5–0.904)	0.788 (0.563–0.974)
ResNet-34	0.886 (0.759–0.984)	0.815 (0.684–0.921)	0.806 (0.643–0.930)	0.834 (0.632–1.00)	0.798 (0.600–0.954)	0.839 (0.647–0.986)
ResNet-34 (CoordAtt)	0.882 (0.748–0.983)	0.842 (0.711–0.947)	0.838 (0.690–0.952)	0.940 (0.818–1.00)	0.762 (0.556–0.947)	0.834 (0.607–0.971)

### 3.4 Classification performance of LC, ROI bounding box, and mask-based ROI model

**Figure 3:**
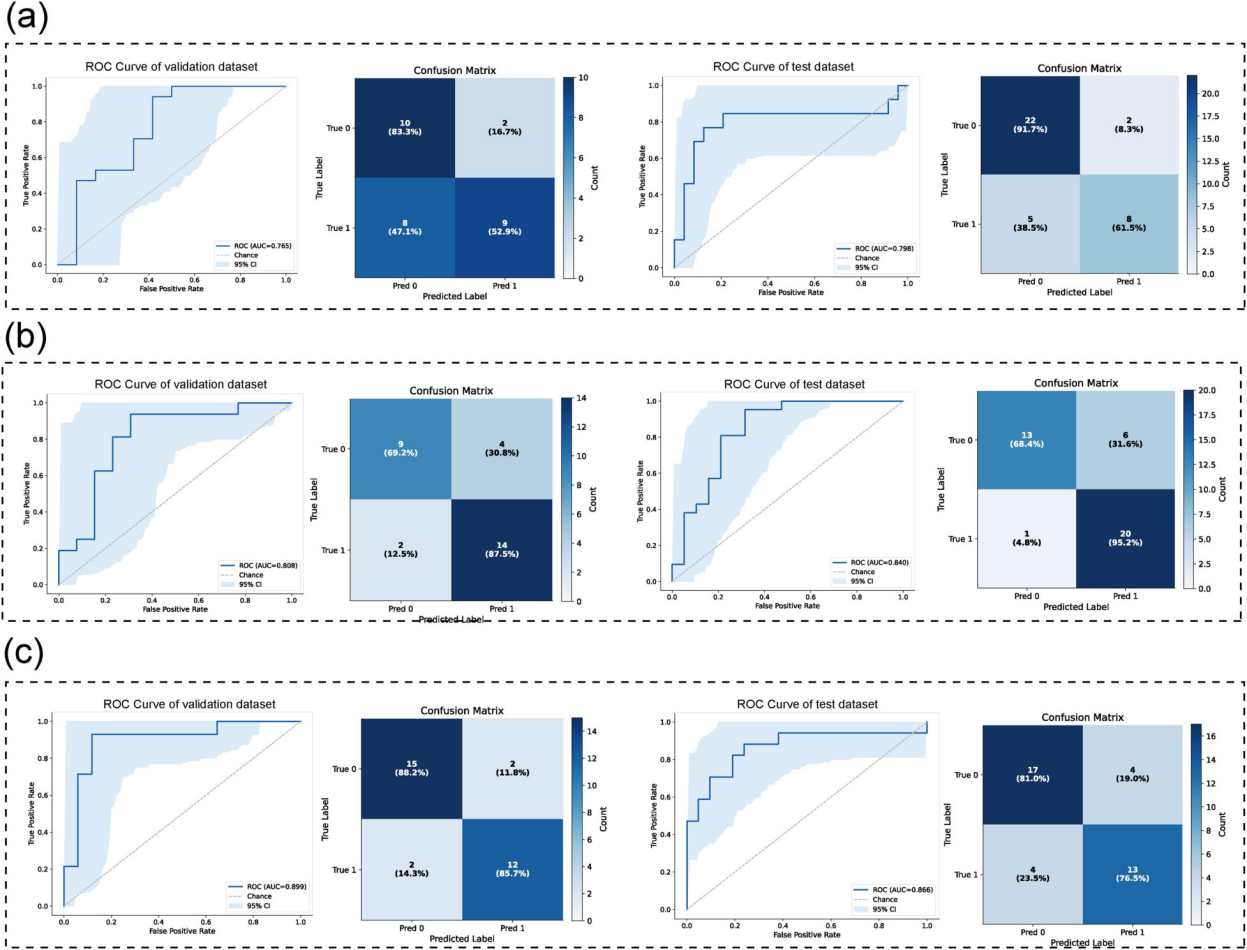
Classification model architecture and performance using different image input strategies. **(a)** Image of LC. **(b)** ROI bounding box of the upper airway. **(c)** Mask-based ROI of the upper airway.

**Figure 4:**
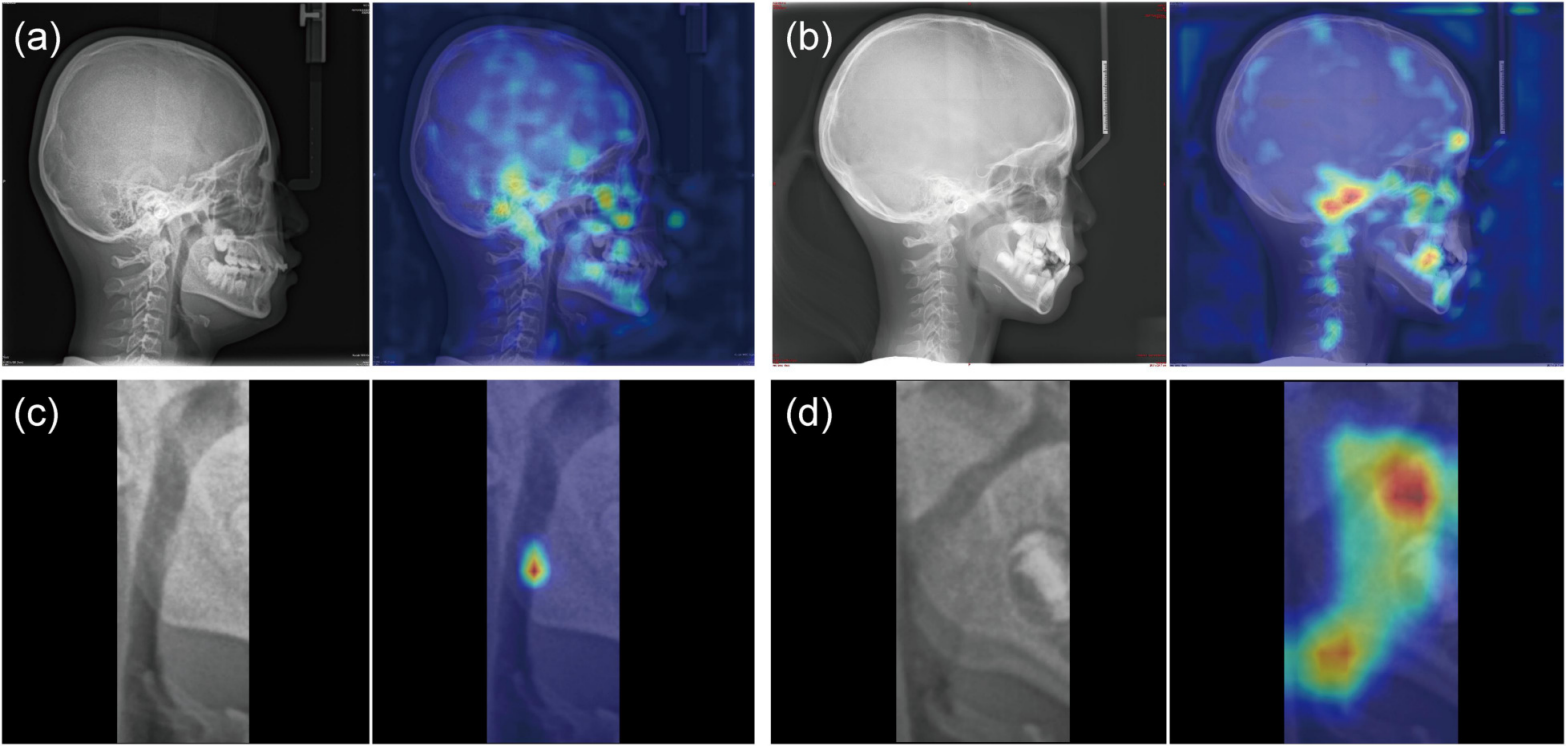
Model explainability **(a)** grad-CAM heatmap for a Non-OSAHS LC. **(b)** Grad-CAM heatmap overlay for an OSAHS patient. **(c)** Grad-CAM heatmap for the airway ROI in a Non-OSAHS case. **(d)** Grad-CAM heatmap for the airway ROI in an OSAHS case.

Table 4 and Figure 3 presents a comparison of models based on different image inputs and regions of interest (ROIs). The model based on the full image achieved an AUC of 0.797 (95% CI: 0.585–0.968), accuracy of 0.811 (95% CI: 0.676–0.918), and an F1 score of 0.683 (95% CI: 0.444–0.882). The ROI-rectangular model demonstrated an improvement, with an AUC of 0.840 (95% CI: 0.702–0.953) and an F1-score of 0.848 (95% CI: 0.731–0.943). Further improvement was observed with the ROI model, which achieved an AUC of 0.882 (95% CI: 0.748–0.983), F1-score of 0.838, and sensitivity of 0.940 (95% CI: 0.818–1.00). These results suggest that ROI-based models outperform the full image models in terms of performance. Figure 4 shows that the Grad-CAM for the full LCs highlights the occlusion and the relationship between the cranial base, maxilla, mandible, occlusion, and cervical spine, whereas the ROI focuses on the airway obstruction areas.

**Table 4:** Evaluation of the performance of LC, bounding-box ROI, mask-based ROI.

Models	AUC (95% CI)	Accuracy (95% CI)	F1-Score (95% CI)	Sensitivity (95% CI)	Specificity (95% CI)	PR-AUC (95% CI)
LC	0.797 (0.585–0.968)	0.811 (0.676–0.918)	0.683 (0.444–0.882)	0.612 (0.333–0.889)	0.917 (0.791–1.000)	0.763 (0.500–0.956)
Bounding-box ROI	0.840 (0.702–0.953)	0.825 (0.700–0.925)	0.848 (0.731–0.943)	0.952 (0.842–1.000)	0.684 (0.461–0.875)	0.823 (0.643–0.964)
Mask-based ROI	0.882 (0.748–0.983)	0.842 (0.711–0.947)	0.838 (0.690–0.952)	0.940 (0.818–1.00)	0.762 (0.556–0.947)	0.834 (0.607–0.971)

### 3.5 Classification performance of fusion model

**Figure 5:**
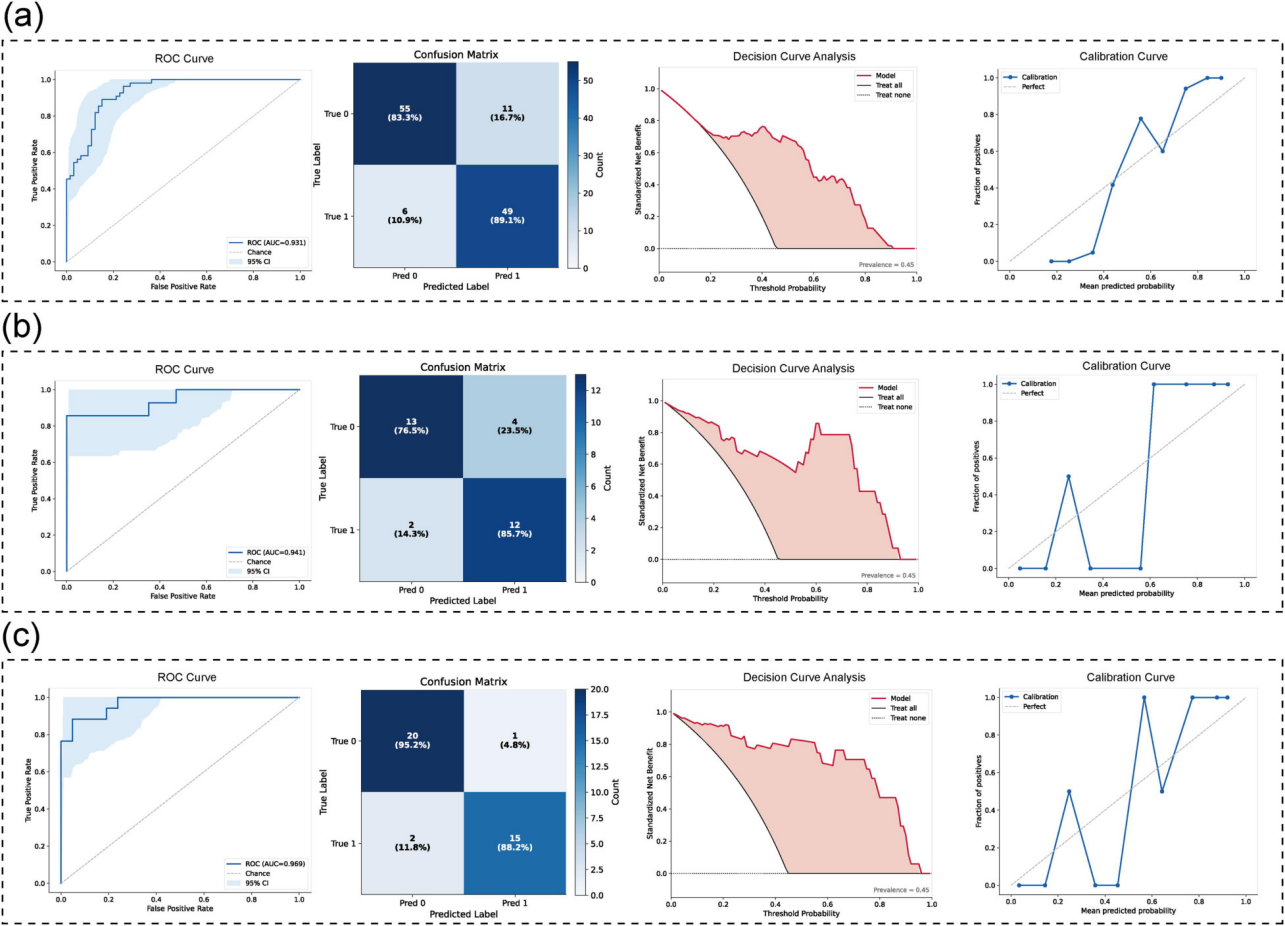
Fusion model evaluation. **(a)** The ROC curve, confusion matrix, DCA and calibration curve of train dataset. **(b)** The ROC curve, confusion matrix, DCA and calibration curve of validation dataset **(c)** the ROC curve, confusion matrix, DCA and calibration curve of test dataset.

Table 5 and Figure 5 presents the performance of the fusion model of LCs and mask-based ROIs. In the training dataset, the fusion model achieved an AUC of 0.931 (95% CI: 0.883–0.967), accuracy of 0.860, and an F1-score of 0.851. In the validation dataset, the model achieved an AUC of 0.940 (95% CI: 0.833–1.000) and an F1-score of 0.792. In the test dataset, the AUC reached 0.945 (95% CI: 0.863–0.994), with an F1-score of 0.824. The decision curve analysis (DCA) and calibration curve in Figure 5 demonstrate that the fusion model exhibits stable performance and high classification accuracy across all datasets.

**Table 5:** Evaluation of the classification performance of fusion model.

Models	AUC (95% CI)	Accuracy (95% CI)	F1-Score (95% CI)	Sensitivity (95% CI)	Specificity (95% CI)	PR-AUC (95% CI)
Train dataset	0.931 (0.883–0.967)	0.860 (0.793–0.917)	0.851 (0.779–0.913)	0.888 (0.796–0.959)	0.836 (0.738–0.915)	0.916 (0.853–0.963)
Validation dataset	0.940 (0.833–1.000)	0.806 (0.645–0.935)	0.792 (0.609–0.938)	0.855 (0.636–1.000)	0.765 (0.545–0.944)	0.951 (0.851–1.000)
Test dataset	0.945 (0.863–0.994)	0.837 (0.711–0.947)	0.824 (0.667–0.941)	0.873 (0.688–1.000)	0.809 (0.615–0.952)	0.933 (0.818–0.995)

### 3.6 Reader study

**Figure 6:**
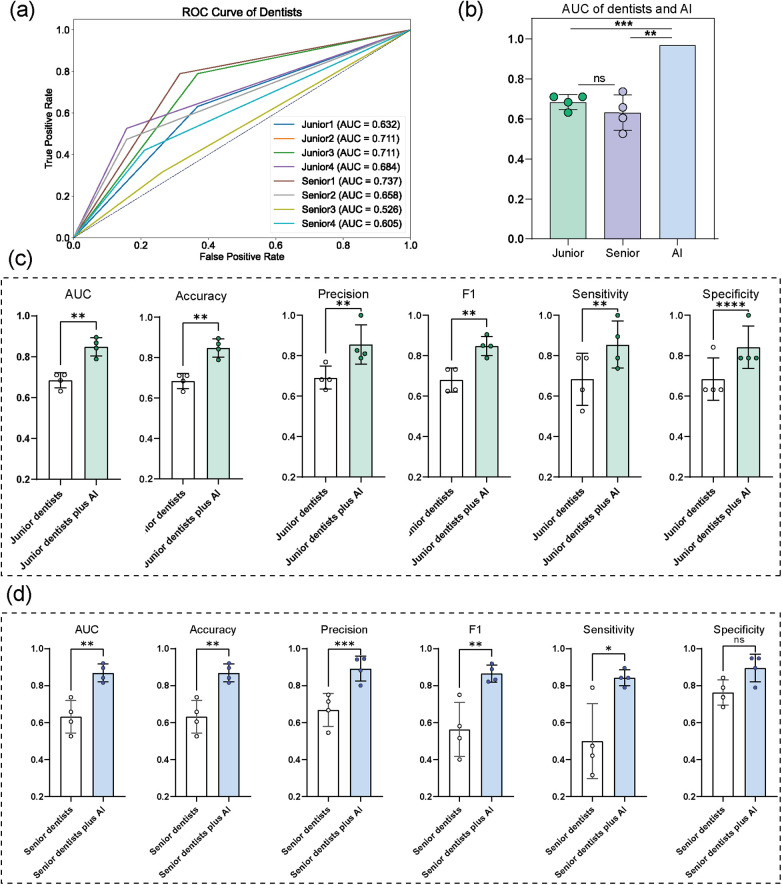
Reader study **(a)** the ROC curve of dentists’ (*n* = 8) performance using LCs alone. **(b)** The AUC of junior dentists, senior dentists and AI **(c)** performance metrics for junior dentists’ performance with and without AI assistance **(d)** performance metrics for senior dentists’ performance with and without AI assistance.

Table 6 and Figure 6 demonstrate the benefits of model improvement for different observers. The AUC for the model was superior to that of both junior and senior dentists, demonstrating significant improvements in diagnostic performance across all observers. With the assistance of AI, the overall diagnostic performance of all dentists showed a noticeable improvement. Specifically, the accuracy for junior and senior dentists increased by 0.165 and 0.237, respectively, following the AI assistance. These results indicate that the use of the model led to significant improvements in diagnostic performance across observers with different experience levels, with more pronounced enhancements observed among senior observers.

**Table 6:** The performance of improvement of reader study with model.

Observer	Accuracy	F1-Score	Sensitivity	Specificity	Precision
Junior1	+0.158	+0.158	+0.158	+0.158	+0.158
Junior2	+0.184	+0.173	+0.211	+0.158	+0.144
Junior3	+0.132	+0.118	+0.105	+0.158	+0.128
Junior4	+0.184	+0.223	+0.211	+0.158	+0.231
Senior1	+0.184	+0.169	+0.105	+0.263	+0.230
Senior2	+0.237	+0.308	+0.368	+0.105	+0.191
Senior3	+0.289	+0.421	+0.526	+0.053	+0.255
Senior4	+0.237	+0.317	+0.368	+0.105	+0.216

## 4 Discussion

Early diagnosis of OSAHS is crucial for improving children's quality of life and preventing potential long-term health issues. While PSG is the gold standard for diagnosis, it faces several limitations, particularly in pediatric populations. This study successfully developed an AI-assisted diagnostic framework based on LC and demonstrated excellent performance in an independent test set (AUC = 0.945, 95% CI 0.863- 0.994; F1 = 0.911, 95% CI 0.667- 0.941), providing a convenient, accurate, and interpretable screening tool for pediatric OSAHS.

In recent years, the application of AI in predicting OSAHS from LC has gradually increased. Jeong et al. and Tsuiki et al. utilized CNNs to diagnose adult OSA, achieving AUCs of 0.82 and 0.92, respectively (22, 23). However, these methods rely solely on shallow regional detection in adults, failing to capture the complex pathological features present in children. Liu et al. proposed an automated method for assessing adenoid hypertrophy using VGG-Lite, which achieved a sensitivity of 0.898 (27). Rao et al. introduced a DL method based on multi-scale local attention for detecting adenoid hypertrophy (25, 26). Despite these promising results, their method did not explore the direct relationship between adenoid hypertrophy and OSAHS. Cai et al. employed DL to evaluate the correlation between adenoid hypertrophy and the AHI, proposing an effective adenoid hypertrophy quantification method with an AUC of 0.85 and a correlation coefficient ranging from 0.9052 (for tonsil size +1) to 0.4452 (for tonsil size +3) (35). However, their study was limited to nasopharyngoscopic images and lacked a comprehensive analysis of other regions of the upper airway. Meanwhile, Su et al. established a predictive model based on craniofacial developmental features of pediatric OSAHS using LASSO regression, achieving an AUC of 0.812 (36). However, the dependence on manual landmarks and measurements may limit efficiency and reproducibility in large-scale screening.

Given the limitations of existing studies, we propose a novel framework that incorporates LCs. The pipeline is fully automated and end-to-end. Compared with existing methods, our model integrates craniofacial and upper airway features to improve the diagnosis performance. The visualization results were anatomically plausible. Grad-CAM activations were frequently observed around the cranial base, maxilla, mandible, occlusal region, and cervical spine. The airway ROI mainly emphasized regions of airway narrowing, including the adenoid and tonsil areas. Prior studies have reported an association between pediatric OSAHS and shortened cranial base length (37, 38). Lymphoid hypertrophy, especially adenoids and tonsils, is also a key contributor to airway obstruction in children (39, 40). The model design reflects these observations. nnU-Net was used for precise airway segmentation. The classifier incorporated a CoordAtt module to strengthen location-sensitive features, which may benefit the analysis of complex airway structures on LCs. On this task, our approach outperformed AlexNet, ResNet-18, and ResNet-34. We also used ImageNet-pretrained weights to initialize the network, which stabilized training and reduced the need for manual feature design.

Furthermore, our model substantially improved the diagnostic performance of dentists. Our model highlighted regions corresponding to the tonsil and adenoid areas, which are crucial in airway narrowing and the pathophysiology of OSAHS. With model assistance, diagnostic accuracy increased by 0.165 for junior dentists and by 0.237 for senior dentists. The larger gain in senior dentists may reflect better use of model cues in borderline or anatomically complex cases. For junior dentists, the tool may help standardize attention to airway-related anatomy and support training. In routine practice, adenoid hypertrophy can be judged quickly in a single case, but large-scale screening is time-consuming and shows inter-observer variability. Automated support may improve efficiency and reduce inconsistency.

Although these results are promising, several limitations remain. First, as a retrospective study, this work is susceptible to inherent selection bias, particularly because some control subjects were identified based on questionnaire screening together with CBCT and clinical evaluation rather than PSG confirmation. As a result, mild or subclinical OSAHS may not have been completely excluded from the control group, which could have introduced label noise and affected model discrimination and generalizability. Second, although patients from two hospitals were included, the sample size remains modest for DL applications. This may have limited the stability of performance estimates and restricted representation of the broader clinical heterogeneity of pediatric OSAHS. Therefore, external validation through prospective multicenter trials will be essential to confirm our results and support clinical translation. Finally, although the model performed well using LCs alone, pediatric OSAHS is also influenced by clinical factors such as age, sex, and obesity, which were not included in the current model. Incorporating these variables in future work may improve predictive performance and strengthen the evidence for clinical implementation, potentially through multimodal integration of imaging and clinical data.

## 5 Conclusion

In this study, we developed and validated an interpretable DL framework for the assessment of pediatric OSAHS using LCs. The model significantly enhanced the diagnostic performance of dentists, enabling them to make timely referrals and improve early detection. To ensure its clinical relevance, future large-scale, multicenter prospective studies are essential to validate the model's generalizability and practical utility.

## Data Availability

The raw data supporting the conclusions of this article will be made available by the authors, without undue reservation.
