# Supplementary material for: Interpretable two-stage deep learning for pediatric obstructive sleep apnea diagnosis using lateral cephalograms
Source: Front Pediatr. 2026 Apr 21;14:1817094. doi: 10.3389/fped.2026.1817094 (PMC13139131; doi:10.3389/fped.2026.1817094)
Supplement: Supplementary file 1 [file Supplementaryfile1.docx]

**Supplementary Materials**

**Interpretable two-stage deep learning for pediatric obstructive sleep apnea diagnosis using lateral cephalograms**

**Jiayi Zhang^1,2,3,4#^, Jiao Tan^1,2,3,4,^ Xuesha Tong^1,2,3,4^, Huiya Wang^1,2,3,4,^ Yue Zhao^6^, Jinlin Song^1,2,3,4*^, Yang Liu^1,2,3,4,5*^**

^1^ The Affiliated Stomatological Hospital of Chongqing Medical University, Chongqing, 401147, China

^2^ Chongqing Key Laboratory of Oral Diseases, Chongqing, 401147, China

^3^ Chongqing Municipal Key Laboratory of Oral Biomedical Engineering of Higher Education, Chongqing, 401147, China

^4^ Chongqing Municipal Health Commission Key Laboratory of Oral Biomedical Engineering, Chongqing, 401147, China

^5^ Western Institute of Digital-Intelligent Medicine，Chongqing 401329, China

^6^ School of Communication and Information Engineering, Chongqing University of Posts and Telecommunications, Chongqing 400065, China.

*** Correspondence:**

Jinlin Song^1,2,3,4*^

E-mail: [songjinlin@hospital.cqmu.edu.cn](mailto:songjinlin@hospital.cqmu.edu.cn)

Yang Liu^1,2,3,4,5*^

E-mail: yangliu@hospital.cqmu.edu.cn

**Table S1. Checklist for Artificial Intelligence in Medical Imaging (CLAIM)**

| Checklist for Artificial Intelligence in Medical Imaging (CLAIM) | | | |
| --- | --- | --- | --- |
| Section/  Topic | No. | Item | Section/ Page and line |
| TITLE | | | |
|  | 1 | Identification as a study of AI methodology, specifying the category of technology used (eg, deep learning) | Title |
| ABSTRACT | | | |
|  | 2 | Structured summary of study design, methods, results, and conclusions | Abstract |
| INTRODUCTION | | | |
|  | 3 | Scientific and clinical background, including the intended use and clinical role of the AI approach | Introduction |
|  | 4 | Study objectives and hypotheses | Introduction |
| Methods | | | |
| Study Design | 5 | Prospective or retrospective study | Material and methods |
|  | 6 | Study goals, such as model creation, exploratory study, feasibility study, noninferiority trial | Material and methods |
| Data | 7 | Data sources | Material and methods |
|  | 8 | Eligibility criteria: how, where, and when potentially eligible participants or studies were identified (eg, symptoms, results from previous tests, inclusion in registry, patient-care setting, location, dates) | Fig 1 |
|  | 9 | Data preprocessing steps | Material and methods -Image Preprocessing and Segmentation |
|  | 10 | Definitions of data elements, with references to common data elements | Material and methods- Patient Data Collection |
|  | 11 | De-identification methods | We removed explicit patient identifiers such as PatientName, PatientID, PatientBirthDate, PatientSex, PatientAge, PatientWeight, PatientAddress, InstitutionName, SOPInstanceUID, Manufacturer, StationName, and so on. |
|  | 12 | How missing data were handled | N/A |
|  | 13 | Definition of ground truth reference standard, in sufficient detail to allow replication | Material and methods- Image Preprocessing and Segmentation |
| Ground Truth | 14 | Rationale for choosing the reference standard (if alternatives exist) | N/A |
|  | 15 | Source of ground truth annotations; qualifications and preparation of annotators | Material and methods-Image Preprocessing and Segmentation |
|  | 16 | Annotation tools | Labelme (version 5.0.1; Computer Science and Artificial Intelligence Laboratory, MIT, Cambridge, MA, USA). |
|  | 17 | Measurement of inter- and intrarater variability; methods to mitigate variability and/or resolve discrepancies | Material and methods-Image Preprocessing and Segmentation |
|  | 18 | How data were assigned to partitions; specify proportions | Material and methods Patient Data Collection |
| Data Partitions | 19 | Detailed description of model, including inputs, outputs, all intermediate layers and connections | Material and methods-Segmentation Model,Classification Model |
|  | 20 | Software libraries, frameworks, and packages | Material and methods The model was trained on an NVIDIA GeForce RTX 4090D GPU with 24 GB of memory using Python 3.10.15. |
|  | 21 | Initialization of model parameters | Transfer learning(pre-trained ImageNet model) |
| Model | 22 | Details of training approach, including data augmentation, hyperparameters, number of models trained | Supplementary material-Table2 |
|  | 23 | Method of selecting the final model | Material and methods  Results-Table2/P |
|  | 24 | Ensembling techniques, if applicable | Material and methods Multi Layer Perceptron (MLP) classifier |
| Training | 25 | Metrics of model performance | Material and methods |
|  | 26 | Statistical measures of significance and uncertainty (eg, confidence intervals) | Confidence intervals |
|  | 27 | Methods for explainability or interpretability (eg, saliency maps) and how they were validated | Material and methods-Interpretability |
| Results | 28 | Demographic and clinical characteristics of cases in each partition | Table 1 |
|  | 29 | Performance metrics for optimal model(s) on all data partitions | Table2,4,5 |
| Discussion | 30 | Implications for practice, including the intended use and/or clinical role | Discussion |
|  | 31 | Study limitations, including potential bias, statistical uncertainty, and generalizability | Discussion |
| Other information | 32 | Where the full study protocol can be accessed | The code and protocol can be offered from the corresponding authors on reasonable request. |
|  | 33 | Sources of funding and other support; role of funders | Acknowledgments & Funding |

**Table S2. Screening criteria for the non-OSAHS control group without PSG**

| **Subject** | **Screening criterion** |
| --- | --- |
| Questionnaire screening | Negative combined screening using PSQ and OSA-18 |
| Adenoidal and tonsillar status on CBCT | No obvious adenoidal or tonsillar hypertrophy |
| Nasal airway on CBCT | Patent nasal airway |
| Upper airway on CBCT | No apparent upper-airway narrowing |
| Record review | No documented clinical history suggestive of sleep-disordered breathing |

**Table S3. Properties of pre-trained CNNs**

| **Network Model** | **Pretrained** | **Parameter(Millions)** | **Size(MB)** |
| --- | --- | --- | --- |
| AlexNet | Yes | 57.0 | 217.5 |
| ResNet-18 | Yes | 11.2 | 43.0 |
| ResNet-34 | Yes | 21.4 | 81.9 |


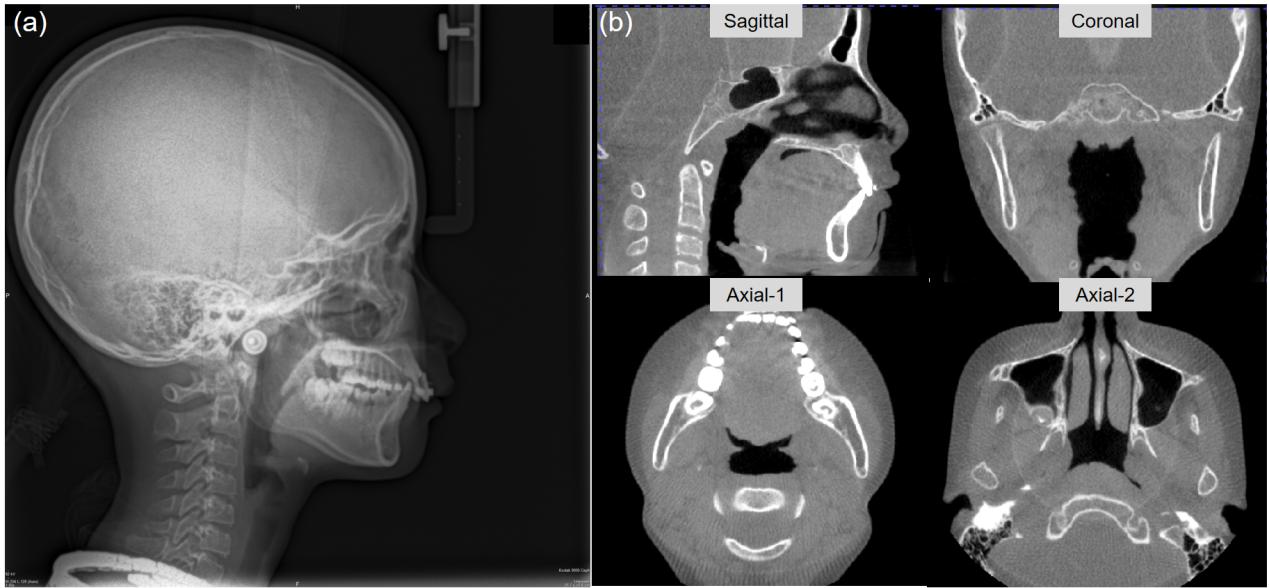


**Figure S1. Representative CBCT images of a non-OSAHS case**
